# Novel Giant Siphovirus from *Bacillus anthracis* Features Unusual Genome Characteristics

**DOI:** 10.1371/journal.pone.0085972

**Published:** 2014-01-27

**Authors:** Holly H. Ganz, Christina Law, Martina Schmuki, Fritz Eichenseher, Richard Calendar, Martin J. Loessner, Wayne M. Getz, Jonas Korlach, Wolfgang Beyer, Jochen Klumpp

**Affiliations:** 1 University of California, Berkeley, Department of Environmental Science, Policy & Management, Berkeley, California, United States of America; 2 Institute of Food, Nutrition and Health, ETH Zurich, Zurich, Switzerland; 3 University of California, Berkeley, Department of Molecular and Cellular Biology, Berkeley, California, United States of America; 4 School of Mathematical Sciences, University of KwaZulu-Natal, Durban, South Africa; 5 Pacific Biosciences, Menlo Park, California, United States of America; 6 University of Hohenheim, Institute of Environmental and Animal Hygiene, Stuttgart, Germany; Rockefeller University, United States of America

## Abstract

Here we present vB_BanS-Tsamsa, a novel temperate phage isolated from *Bacillus anthracis,* the agent responsible for anthrax infections in wildlife, livestock and humans. Tsamsa phage is a giant siphovirus (order *Caudovirales*), featuring a long, flexible and non-contractile tail of 440 nm (not including baseplate structure) and an isometric head of 82 nm in diameter. We induced Tsamsa phage in samples from two different carcass sites in Etosha National Park, Namibia. The Tsamsa phage genome is the largest sequenced *Bacillus* siphovirus, containing 168,876 bp and 272 ORFs. The genome features an integrase/recombinase enzyme, indicative of a temperate lifestyle. Among bacterial strains tested, the phage infected only certain members of the *Bacillus cereus* sensu lato group (*B. anthracis*, *B. cereus* and *B. thuringiensis*) and exhibited moderate specificity for *B. anthracis*. Tsamsa lysed seven out of 25 *B. cereus* strains, two out of five *B. thuringiensis* strains and six out of seven *B. anthracis* strains tested. It did not lyse *B. anthracis* PAK-1, an atypical strain that is also resistant to both gamma phage and cherry phage. The Tsamsa endolysin features a broader lytic spectrum than the phage host range, indicating possible use of the enzyme in *Bacillus* biocontrol.

## Introduction

We present a novel temperate phage isolated from *Bacillus anthracis*, the causative agent of anthrax infections in wildlife, livestock and humans [1]. *Bacillus anthracis* is a member of the *Bacillus cereus* sensu lato group of six closely related species: *B. cereus*, *B. anthracis*, *B. thuringiensis*, *B. mycoides*, *B. pseudomycoides* and *B. weihenstephanensis* [2]. This group contains both obligate and opportunistic animal pathogens, including *B. anthracis*, *B. cereus,* strains of which cause food poisoning and opportunistic infections in humans [3], and *B. thuringiensis,* an insect pathogen commonly used as a pesticide in agriculture [4]. Genomic studies have identified a number of putative prophages in the *Bacillus cereus* sensu lato group (e.g., [5], [6]), including four unique prophage elements in *B. anthracis* [7].

Lysogeny occurs commonly in *B. anthracis* [8] and may play an essential role in its life cycle [9]. Isolates of *B. anthracis* from soil frequently exhibit phage-derived plaques upon subculture [10]. Schuch et al. [9] showed that temperate phage infections of *B. anthracis* can affect sporulation, induce biofilm formation and promote colonization of earthworms and environmental reservoirs. Furthermore, the lytic activity and high specificity of bacteriophages provide a promising resource for the development of innovative treatments for human pathogens, including *B. anthracis*.

In this study, we describe the genome and host range of vB_BanS-Tsamsa, a novel temperate phage obtained from *B. anthracis* isolated in Etosha National Park (Etosha), Namibia. We named the phage Tsamsa, which in Hai||om means “place where the winds blow closed” referring to the endless vista of the Etosha pan and the dust devils that form there. Tsamsa phage is a giant siphovirus capable of infecting some members of *Bacillus cereus* sensu lato.

## Materials and Methods

### Phage Isolation and Preparation

We obtained isolates of the siphovirus from two carcass sites in Etosha, a 22,915 km^2^ national park in northern Namibia with abundant wildlife populations that exhibit regular occurrences of anthrax infections (reviewed in [11]). Field sampling was authorized by the Namibian Ministry of Environment and Tourism under permit number 1448/2009 to HHG. Bacteriophages were isolated from a *B. anthracis* isolate obtained from a swab of a plains zebra (*Equus quagga*) carcass from 2006 (Etosha Ecological Institute (EEI) carcass number: EB060318-01WV, GPS coordinates: −18.99736, 15.81584) and from soil collected near another plains zebra carcass in 2010 (EEI carcass number: EB100228-01MK, GPS coordinates: −19.1731, 15.92603). Prior diagnostic testing showed that both carcasses were positive for *B. anthracis*: isolates from the two carcasses were identified as genotype 6 and genotype 4 in the A cluster of *B. anthracis*, respectively [12]. Genotypes 6 and 4 are closely related members of a dominant *B. anthracis* strain that has been causing outbreaks in Etosha for a very long time [12].

Phages were obtained from the two samples by culturing to enrich for bacteria and by exposure to mitomycin C to induce prophages in the host genome to transition into a vegetative state. Methods for enrichment culture and induction are described by Sambrook [13] and Van Twest and Kropinski [14]. We did not obtain phages from either sample without induction. We used two approaches, one for the swab isolate and another for the soil sample, as follows:

1. For the swab isolate, we inoculated 3 ml of Bovine Heart Infusion medium (BHI, BD Difco, Sparks, MD, USA) with a single *B. anthracis* colony isolated from the swab and incubated the culture overnight at 37°C with aeration. We diluted the overnight culture 100-fold in 3 ml of BHI and incubated it at 37°C with aeration for one hour. To induce the release of prophages from the genome, mitomycin C was added to achieve a final concentration of 2.5 µg ml^−1^. The culture was incubated at 37°C with aeration for 20 hrs and pelleted for 15 min at 3000×*g*. The supernatant was filtered through a 0.22 µm filter unit and stored at 4°C.
2. For the soil sample, 20 g of soil were added to 15 ml of 1% Nutrient Broth medium (BD Difco, Sparks, MD, USA). After vortexing briefly, the sample was incubated at 35°C with aeration overnight. The resulting culture was centrifuged and the supernatant collected. Then we added 1% Nutrient Broth medium to attain a final volume of 7.5 ml. The sample was incubated in 1 µg ml^−1^ mitomycin C for 30 minutes with gentle aeration at 30°C. Then the sample was filtered (0.22 µm) and concentrated using a Vivaspin 20 concentrator (Sartorius Stedim, Bohemia, NY, USA) by adding 10 ml of 1% nutrient broth medium and centrifuging the sample at 3000×*g* for 10 min. The resulting phage extract was stored at 4°C.

### Preparation of Plate Stocks of the Two Tsamsa Phage Isolates

Phage preparations were purified and concentrated using standard techniques [13], [15]. Preliminary plaque assays were performed with phage extracts from the two carcass site samples (swab isolate and soil sample) to harvest concentrated plate stocks. Soft agar overlays were performed as described previously by Adams [16]. Briefly, five microliters of a spore preparation of an avirulent (pXO1− pXO2−) *B. anthracis* strain (6602 R1,[17]) were added to 2.5 ml of LB soft agar (BD Difco, Sparks, MD, USA; containing per liter: 10 g of tryptone, 7 g of agar, 5 g of yeast extract and 5 g of NaCl) and poured over the surface of pre-warmed plates (containing per liter: 8 g of Nutrient Broth, 5 g of NaCl, 15 g of agar, 0.15 g of CaCl_2_, 0.2 g of MgSO_4_ and 0.05 g of MnSO_4_ [18]). After the soft agar solidified, ten microliters of 10^−4^, 10^−6^ and 10^−8^ dilutions of each of the two phage extracts were pipetted on top of each plate and the plates were incubated overnight at 30°C. We selected a single plaque from each of the two phage extract plates and stored it in 0.5 ml of phage buffer (10 mM Tris HCl (pH 8), 10 mM MgCl_2_ and 200 mM NaCl). Dilutions of the phage plaque buffer were added to *B. anthracis* strain 6602 R1 [17] spore preparation in 2.5 ml of LB soft agar and soft agar overlays were performed. Plates with complete clearance were harvested by adding 5 ml of phage buffer to each plate and collecting the soft agar overlays with cell scrapers into 50 ml centrifuge tubes. We centrifuged the tubes at 3000×*g* for 8 min and syringe filtered the supernatant through a 0.22 µm filter unit. The resulting plate stocks were further concentrated using PEG-6000 precipitation [19], followed by cesium chloride density gradient centrifugation to obtain pure phage particles [13] [15] and stored at 4°C for additional analyses.

### Phage Host Range

Tsamsa phage was originally propagated on *B. anthracis* 6602 R1 (an avirulent strain that lacks both pXO1 and pXO2 virulence plasmids, [17]). Using the spot-on-the-lawn method and a 100-fold and a 10,000-fold dilution of the phage stock, 10 µl were spotted on top of plates seeded with a lawn of bacteria. Each plate was tilted to allow the phage solution to run down the plate. If phage titers were higher than 10^8^ pfu/ml, more dilutions were made. A phage was considered positive for infection of a certain strain if single plaques could be observed on one of the spots. We tested a set of 55 strains for susceptibility to Tsamsa phage (Table 1).

**Table 1:** Host range of Tsamsa phage on 43 different *Bacillus* strains and 12 non-*Bacillus* strains and lytic spectrum of Tsamsa endolysin for a subset of the *Bacillus* strains. Presence of lysis is indicated by+and absence of lysis is indicated by -. n.d.: not determined.

Strain name	Organism	Notes	Source	Phage lysis	Endolysin lysis
6602 R1	*Bacillus anthracis*	pXO1-pXO2 negative	[39]	**+**	**+**
Sterne	*Bacillus anthracis*	pXO2 negative	Institut Pasteur #7702	**+**	**+**
Weybridge UM44	*Bacillus anthracis*	pXO2 negative	[39]	**+**	**+**
Ames-non reverting	*Bacillus anthracis*	pXO2 negative	U.S. Dept. of Agriculture, Ames, Iowa	**+**	**+**
Ames	*Bacillus anthracis*		U.S. Dept. of Agriculture, Ames, Iowa	**+**	**n.d**
Vollum 1b	*Bacillus anthracis*		Laboratory Strain	**+**	**n.d.**
PAK-1	*Bacillus anthracis*		Pakistan isolate, M. Hugh-Jones collection	**−**	**n.d.**
569	*Bacillus cereus*		[17]	**+**	**n.d.**
LA 925	*Bacillus cereus*		CHUV	**−**	**+**
ATCC 14579	*Bacillus cereus*		ATCC	**−**	**+**
ATCC 11778	*Bacillus cereus*		ATCC	**+**	**+**
ATCC 10702	*Bacillus cereus*		ATCC	**−**	**+**
ATCC 10876	*Bacillus cereus*		ATCC	**+**	**n.d.**
DSM 2302	*Bacillus cereus*		DSM	**−**	**+**
BO 366	*Bacillus cereus*		This study	**−**	**−**
BO 372	*Bacillus cereus*		This study	**−**	**−**
BO 493	*Bacillus cereus*		This study	**−**	**−**
DSM 4218	*Bacillus cereus*		DSM	**−**	**+**
ATCC 33019	*Bacillus cereus*		ATCC	**+**	**+**
ATCC 14737	*Bacillus cereus*		ATCC	**+**	**+**
DSM1274	*Bacillus cereus*		DSM	**−**	**+**
ATCC 27522	*Bacillus cereus*		ATCC	**−**	**+**
NCTC 11143	*Bacillus cereus*		NCTC	**−**	**+**
NCIMB 8705	*Bacillus cereus*		NCIMB	**+**	**+**
ATCC 6464	*Bacillus cereus*		ATCC	**−**	**+**
B346	*Bacillus cereus*		Mouse isolate	**−**	**+**
DSM360	*Bacillus cereus*		DSM	**−**	**−**
HER1399	*Bacillus cereus*		HER	**+**	**+**
WSBC 10530	*Bacillus cereus*		WSBC	**−**	**−**
WSBC 10556	*Bacillus cereus*		WSBC	**−**	**+**
WSBC 10566	*Bacillus cereus*		WSBC	**−**	**−**
WSBC 10583	*Bacillus cereus*		WSBC	**−**	**−**
DSM4421	*Bacillus thuringiensis*		DSM	**−**	**+**
WSBC 10204	*Bacillus thuringiensis*		WSBC	**−**	**−**
HER1211	*Bacillus thuringiensis*		HER	**+**	**+**
Kurstaki	*Bacillus thuringiensis*		Industry isolate	**−**	**−**
ATCC 10792	*Bacillus thuringiensis*		ATCC	**+**	**+**
DSM168	*Bacillus subtilis*		DSM	**−**	**−**
DSM675	*Bacillus subtilis*		DSM	**−**	**−**
ATCC 23059	*Bacillus subtilis*		ATCC	**−**	**n.d.**
DSM395	*Bacillus sphaericus*		DSM	**−**	**−**
DSM90	*Bacillus megaterium*		DSM	**−**	**−**
WSBC 10550	*Bacillus weihenstephanensis*		WSBC	**−**	**+**
WSLC 3009	*Listeria ivanovii*		WSLC	**−**	
ATCC BAA-679	*Listeria monocytogenes*		ATCC	**−**	
PSK	*Staphylococcus aureus*		Laboratory Stock	**−**	
Twort	*Staphylococcus aureus*		Laboratory Stock	**−**	
414	*Staphylococcus epidermidis*		Laboratory Stock	**−**	
100655	*Staphylococcus epidermidis*		Laboratory Stock	**−**	
602	*Staphylococcus epidermidis*		Laboratory Stock	**−**	
DT7155	*Salmonella* Typhimurium		Laboratory Stock	**−**	
CGSC 4401	*Escherichia coli*		CGSC	**−**	
DSM 20560	*Streptococcus salivarius*		DSM	**−**	
NZ9000	*Lactococcus lactis*		Laboratory Stock	**−**	
ATCC 19433	*Enterococcus faecalis*		ATCC	**−**	

Source abbreviations: CHUV: Strain collection of the Centre Hospitalier universitaire Vaudois, Switzerland; HER: Félix d’Hérelle Reference Center for bacterial viruses, Laval, Canada; ATCC: American Type Culture Collection; NCTC: National Collection of Type Cultures; DSM: Deutsche Sammlung von Mikroorganismen; NCIMB: National Collection of Industrial Bacteria; WSBC: Weihenstephan Bacillus Collection; WSLC =  Weihenstephan Listeria Collection; CGSC: Coli Genetic Stock Center, Yale, USA.

### Phage Morphology

The Tsamsa phage was negatively stained with 2% uranyl acetate on carbon-coated copper grids (Carbongrids, Quantifoil, Jena, Germany) and observed in a Philips CM12 TEM microscope at 120 kV acceleration voltage with a Gatan Orius 1 k camera.

### Genome Sequencing and Genetic Characteristics

We performed a standard DNA extraction using phenol-chloroform-isoamyl-alcohol of the cesium chloride purified stock [13]. After washing with 70% ethanol and drying, the DNA pellet was resuspended in sterile ultrapure water. We sequenced DNA from the two isolates using a SMRT sequencing approach (Pacific Biosciences RS) with 10 kb and 800 bp insert libraries (C2 chemistry) and one SMRT sequencing cell for each library. We used the standard error-correction workflow and SMRT portal software 1.3.1 for assembly of 36166 post-filter reads (with 2582 bp average read length). Open reading frames were predicted by RAST [20] and edited manually. The genome sequence was deposited at GenBank under accession number KC481682. The unassembled reads for both sequencing runs are available in the DNA Databank of Japan Sequence Read Archive under accession number DRA001229.

### Phylogenetic Analysis

Comparisons were made between sequences of the large terminase subunit of Tsamsa phage and 17 other *Bacillus* phages (GenBank accession numbers: 955214, 955254, 7070024, 12980149, 13164871, 14697218, 14697335, 14697413, 14697831, NC_001884, NC_006557, NC_007457, NC_007458, NC_007734, NC_007814, NC_011167, NC_011421). Alignments and phylogenetic tree construction were performed in Geneious version 6.1 (Biomatters Ltd., http://www.geneious.com). Muscle [21] was used to align the terminase gene (with 16 iterations). Then MrBayes 3.1.2 [22], [23] was used to build the tree and determine Bayesian posterior probabilities (with the Monte Carlo Markov Chain run for 1.1×10^6^ generations).

### Endolysin Production and Determination of Lytic Spectrum

Purified Tsamsa phage endolysin was recombinantly produced [24] and characterized. Lysin activity against different bacterial strains was tested with a turbidity reduction assay or plate lysis. Turbidity reduction experiments were performed by harvesting and washing an overnight bacterial culture (grown in half-strength BHI medium) in PBS (120 mM NaCl, 50 mM NaH_2_PO_4_, pH 8.0) buffer. Cell density was adjusted to OD_600nm_ of 1±0.05. 100 µM of lysin were added to the wells of a 96-well plate and 200 µl bacterial suspension was added. Wells were measured at a wavelength of 600 nm in 10 second intervals until clear turbidity reduction was observed relative to the control.

Lysis activity by plate lysis assay was tested as follows. The *Bacillus* strains were grown in half-strength BHI medium to an OD_600nm_ of 0.4–0.6 and diluted 1/100 in PBS immediately before plating. The freshly spread lawns of *Bacillus* cells on ½ BHI agar plates were air dried for 30 min. Ten microliters of a 10-fold serial dilution (50, 5 and 0.5 µM) of the purified endolysin were spotted onto the plates. Spots were air-dried and plates incubated at 30°C for 16 hours. Cleared spots indicating cell lysis were assessed visually.

## Results

We obtained two phage isolates from two different carcass sites (one from soil and one carcass swab). Both phages were morphologically very similar and unusually large compared with previously described *Siphoviridae* of *Bacillus*. We found by sequencing the genomes of these two isolates that both phages were 100% identical. The representative phage was named Tsamsa and its characteristics were investigated further.

### Tsamsa Features a Broad Host Range in the *B. anthracis* Subgroup

A set of 55 bacterial strains was analyzed for susceptibility to Tsamsa. The phage lysed seven out of 25 *B. cereus* strains as well as two out of five *B. thuringiensis* strains and six out of seven *B. anthracis* strains (6602 R1, Sterne, Weybridge UM44, Ames-non-reverting and Vollum 1b but not PAK-1) (Table 1). It should be noted that PAK-1 is an atypical member of *B. anthracis*, belonging to the A2 branch that contains very few isolates [25], and is resistant to both Cherry phage and Gamma phage [26]. Tsamsa phage is unable to infect *B. subtilis* 168 and DSM675, *B. megaterium* DSM90, *B. sphaericus* DSM395, *B. weihenstephanensis* WSBC10550 as well as all tested strains from other bacterial genera (*Listeria ivanovii* and *L. monocytogenes*, *Staphylococcus aureus* and *S. epidermidis*, *Salmonella* Typhimurium, *Escherichia coli*, *Streptococcus salivarius*, *Lactococcus lactis* or *Enterococcus faecalis*) (Table 1). Thus it is a narrow host-range virus that infects some members of the *Bacillus cereus* sensu lato group and exhibits moderate specificity for *B. anthracis*.

### Tsamsa is a Giant Siphovirus

**Figure 1:**
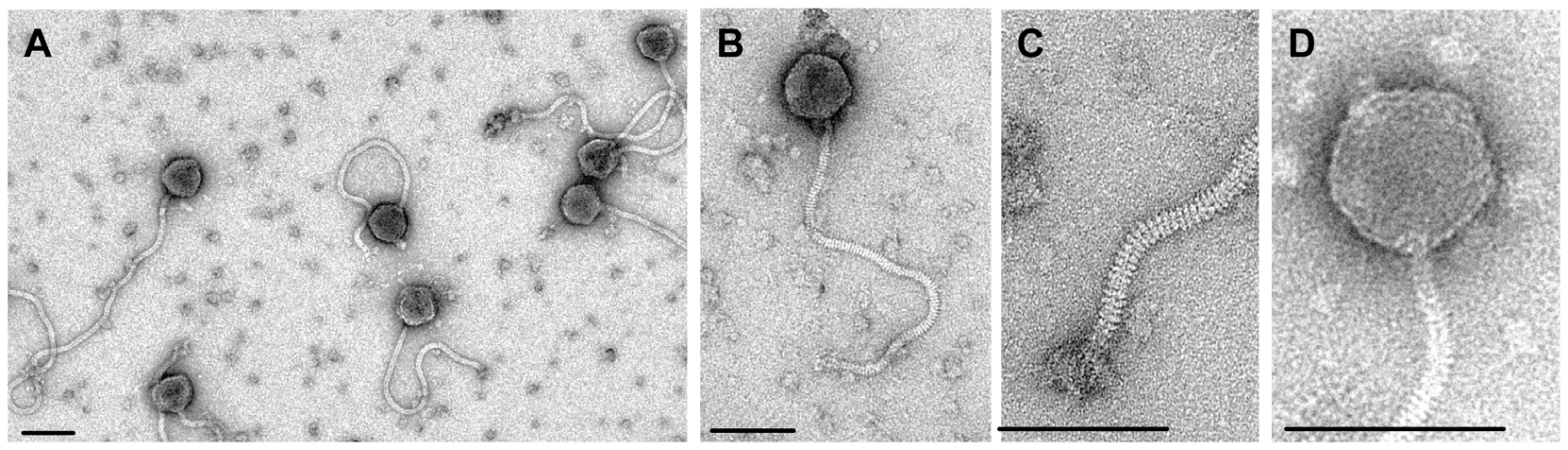
Electron microscopy of Tsamsa phage. TEM images were acquired from a preparation of pure phage particles negatively stained with 2% uranyl acetate on carbon-coated copper grids (Quantifoil, Jena, Germany) and observed using a Philips CM12 microscope at 120 kV acceleration voltage with a Gatan Orius digital camera. **A.** Preparation overview. **B.** Close-up of single phage particle. **C.** Details of the phage tail distal end. **D.** Details of the phage head structure. Individual capsomers are visible, an observation previously made for SPO1-related phages ([27]). Scale bars represent 100 nm.

Most bacteriophages belong to the Order *Caudovirales*, which contains three families: *Myoviridae* with a contractile tail, *Siphoviridae* with a non-contractile flexible tail and *Podoviridae* with a short, non-contractile tail. Tsamsa exhibits typical siphovirus morphology, featuring a long, flexible and non-contractile tail of 440 nm (not including baseplate structure) and an isometric head of 82 nm in diameter (Figure 1 A and B). Individual tail striations (disk-like structure) and a baseplate structure with appendages are visible (Figure 1C). The head features visible individual capsomers (Figure 1D), an observation previously made for a different class of large virulent phages belonging to the *Spounavirinae* subfamily within the family *Myoviridae* [27]. Because of the large head dimensions and our experience from similarly sized myoviruses, we anticipate a triangulation number of 16 or higher but experimental proof is lacking.

### The Phage Tsamsa Genome is Large and Unique

**Figure 2:**
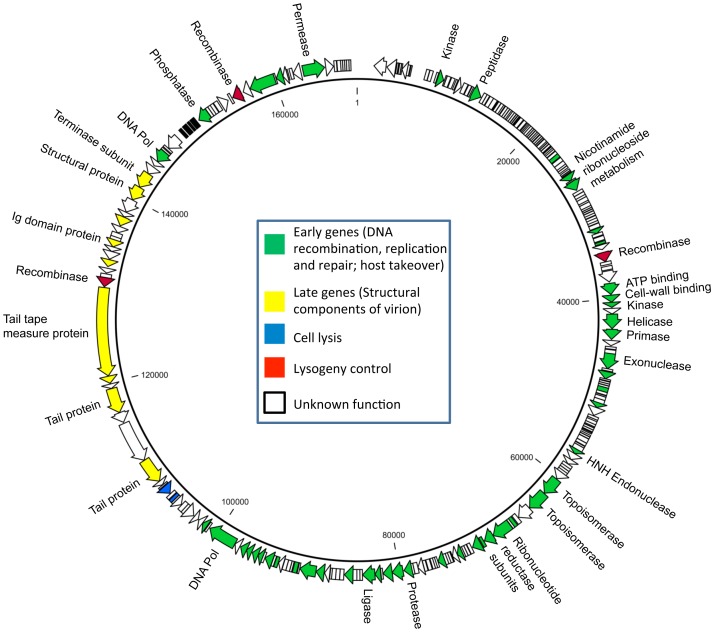
Genome map of Tsamsa phage. Open reading frames are drawn to scale and transcription direction is indicated by arrows. Selected proteins with putative function are labeled. Genetic modules (i.e. structural genes, early genes) are indicated by coloring.

**Figure 3:**
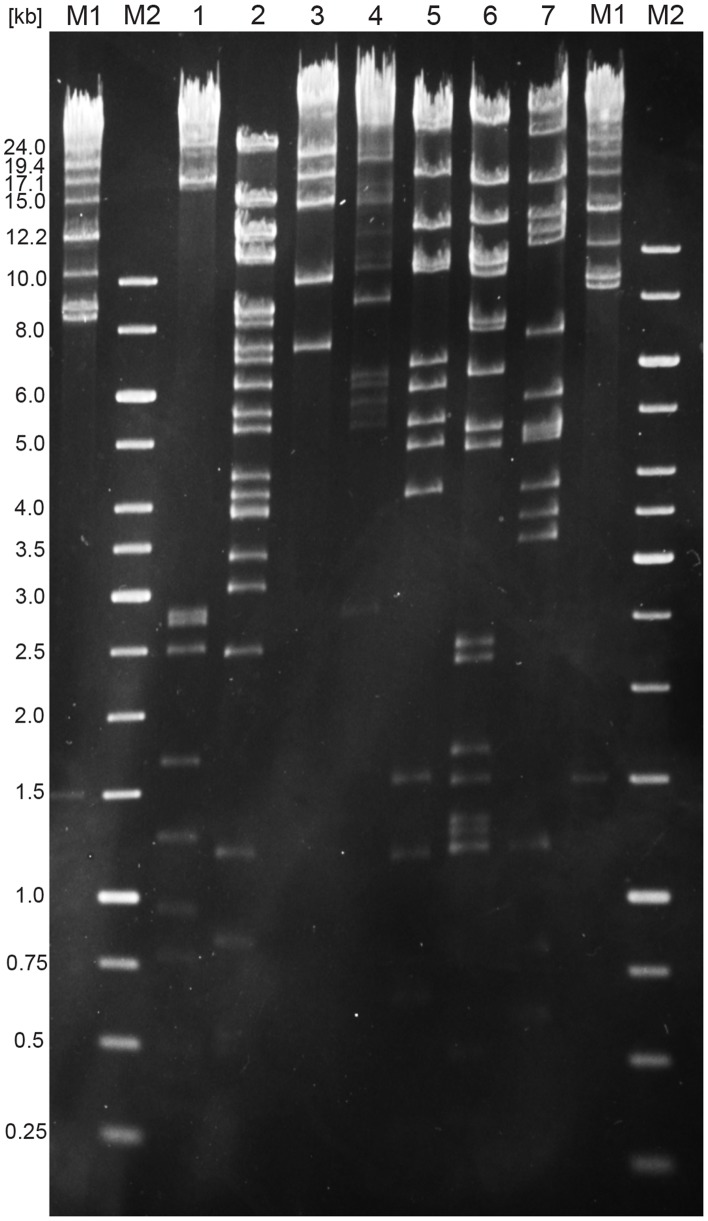
Restriction profile of Tsamsa phage. 500 ng DNA were digested with different restriction enzymes for 2 h at 37°C and electrophoresed. Clear band separation up to 24 kb in size was achieved and the restriction profiles matched with the sequenced genome size. The terminal redundancy location and size was determined from the fragment sizes as previously described [13], [15]. Enzymes used: 1: Alw44I (NEB); 2: Eco91I (Fermentas); 3: NheI (NEB); 4: PacI (NEB); 5: SwaI (NEB); 6: Van91I (Fermentas); 7: XcmI (NEB). M1: Lambda 19 Mix Size standard (Fermentas); M2: 1 kb size standard (Fermentas). Numbers to the left indicate band size in kb.

Genome sequencing and assembly resulted in a single large contig with an average coverage of 550-fold of error-corrected SMRT reads (Figure 2). Both phage isolates were identical. A repeat structure of 284 bp at both genome ends was identified during assembly and confirmed in restriction profiles (Figure 3). Methylome analysis revealed no base modifications in the genome. The genome sequence is 168,876 bp in length. Tsamsa features 272 open reading frames, 17 tRNA and 2 pseudo-tRNA genes. Database matches of predicted proteins encoded by *Bacillus anthracis* phage Tsamsa are provided in Table S1. The GC content is 34%, similar to published genome sequences of *B. anthracis*.

### Virus Proteome

Virion proteins of Tsamsa were separated on a 10–20% SDS gradient PAGE. Resulting bands were extracted and protein content identified by mass spectrometry [28]. Six bands were allocated to gene products. The tape measure protein is present in two protein bands of 280 and 100 kDa in size, presumably because of instability of the large protein or post-translational modification. gp206 was identified in a band with an estimated mass of 38 kDa and gp199 and 207 were identified in bands of 26.5 and 19 kDa, respectively.

### Comparison with Other Sequenced *Bacillus* Phages

**Figure 4:**
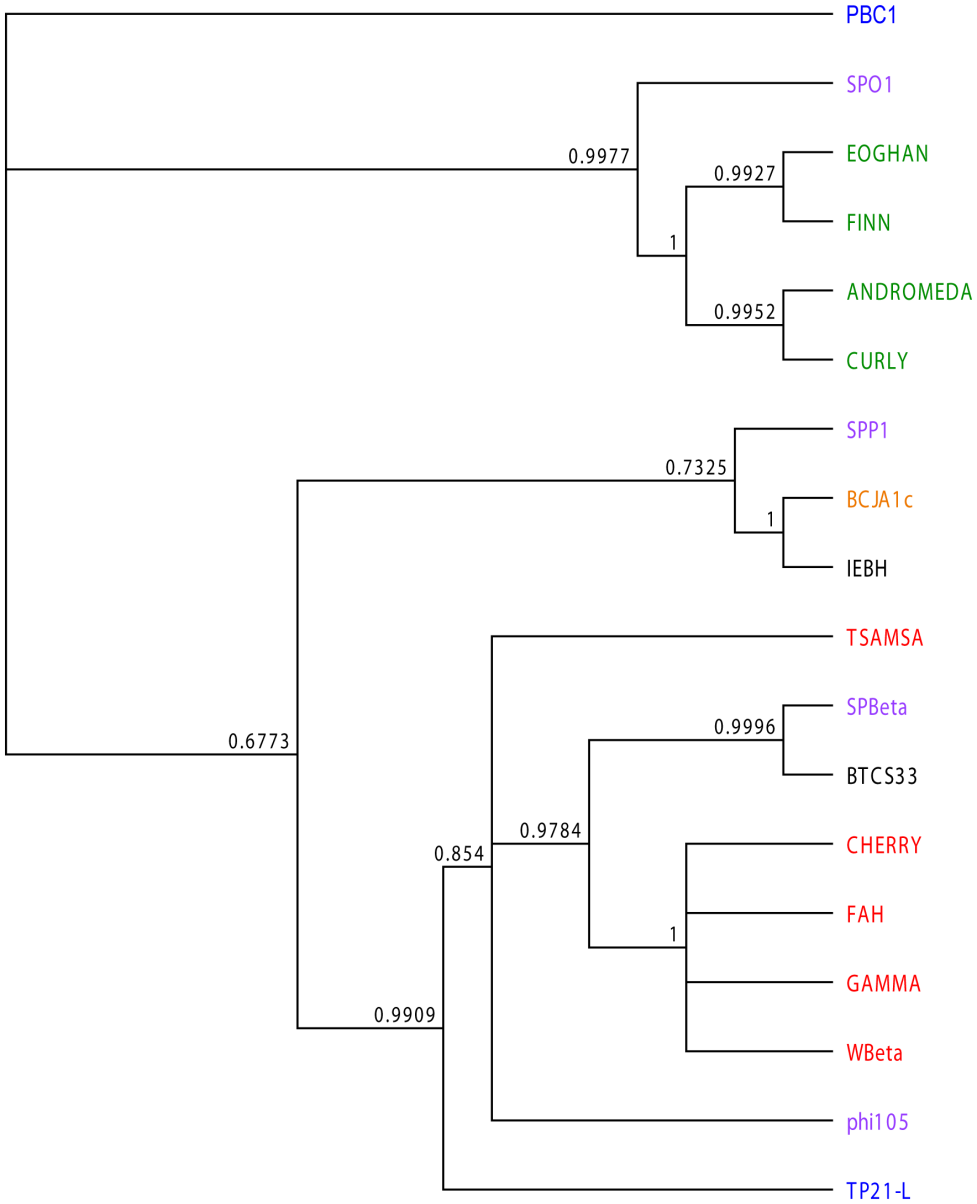
Phylogenetic relationship of the large terminase subunit gene in 18 *Bacillus* phages. Phages are color coded according to their host bacterium: *B. anthracis* in red, *B. cereus* in blue, *B. clarkii* in orange, *B. pumilus* in green, *B. subtilis* in purple and *B. thuringiensis* in black. The tree and posterior probabilities were determined from 1.1x10^6^ generations with MrBayes 3.1.2 [22], [23].

The sequence of the large terminase subunit of Tsamsa phage was compared with 17 previously sequenced *Bacillus* phages: four *B. anthracis* phages (Fah, Cherry, WBeta, Gamma), two *B. thuringiensis* phages (IEBH, BTCS33), four B. *subtilis* phages (SPBeta, SPO1, SPP1, phi105), one *B. clarkii* phage (BCJA1c), two *B. cereus* phages (PBC1, TP21-L) and four *B. pumilus* phages (Andromeda, Curly, Eoghan, Finn). The Tsamsa phage terminase clearly differs from previously described phages isolated from *B. anthracis* (Figure 4), which were shown to be derived from a single *B. anthracis* prophage named W [29], [30].

### The Tsamsa Endolysin Shows Broad Activity

The endolysin of phage Tsamsa (gp217) was cloned and recombinantly produced in *E. coli*. The 35.78 kDa protein features an isoelectric point of 9.01 and contains three domains, one Amidase_2 enzymatic active domain (PF01510) and two SH3_3 (PF08239) cell-wall binding domains. Lytic activity of the protein was assessed with either turbidity reduction or plate lysis assay and activity against a set of *Bacillus* strains is outlined in Table 1. The Tsamsa endolysin features a broad spectrum of lytic activity and is able to lyse more *Bacillus cereus* sensu lato strains than the phage can infect.

## Discussion

We obtained identical phage isolates of an unusually large siphovirus induced from *B. anthracis* in two different environmental samples. Such a large siphovirus is rarely isolated; only nine of the 539 Siphovirus genomes in the NCBI Genome database have a larger genome than Tsamsa phage (six *Caulobacter* phages, two *Synechococcus* phages and one *Erwinia* phage). To our knowledge, Tsamsa is the largest sequenced siphovirus infecting *Bacillus*. Two larger *Bacillus* siphoviruses are known but not characterized: *B. mycoides* phage N5 and *B. thuringiensis* phage II, both of which feature approximately 20% larger dimensions than Tsamsa and are speculated to be identical (H.-W. Ackermann, personal communication). Large siphoviruses may be isolated infrequently due to a bias in phage isolation procedures towards smaller phages. Consequently, the distribution and abundance of large siphoviruses are poorly understood. Such a sampling bias has been reported for the so-called Jumbo-Phages [31].

Like many siphoviruses, the genome is structured in functional modules. The early gene cluster (genes for DNA replication, modification and repair, host takeover and nucleotide metabolism) spans roughly 70% of the genome, indicating active participation of virus-encoded genes in the metabolic processes associated with replication in the host cell. It is notable that the Tsamsa genome encodes three tyrosine integrase/recombinase enzymes of the Cre/XERD type (gp94, gp227, gp255; [32], [33], which exhibit no homology to each other. Tsamsa features a temperate lifestyle and these three enzymes may serve as means to integrate into different *attB* sites and ensure a large host range for lysogeny. Further work will elucidate the specificity and activity of the three recombinases. We also note the presence of two Ig-domain containing proteins, gp233 and gp213 [33], [34], which may play accessory roles during infection [35]. Ig-like domains in structural proteins were recently shown to play a potential role in phage attachment to mucosa of humans and animals by interaction of the Ig-like domain with the mucosa glycan residues, providing a non-host derived immunity [36].

Tsamsa features distributed homologies in its structural proteins to SPO1-related phages: A511, A9, LP65 and SPO1 [27]. This finding is very unusual because SPO1-related phages belong to the *Spounavirinae* subfamily within an unrelated family of bacteriophages (*Myoviridae*) [27]. In addition, Tsamsa is a temperate phage and phages within the *Spounavirinae* are strictly virulent. Tsamsa also displays individual capsomers thought to be a hallmark of the *Spounavirinae* (Figure 1) [27]. An assessment of the virus particle proteome identified six structural protein bands in Tsamsa, namely the tape measure protein in two bands, and one band each for gp199, 206, 207 and 222. Tsamsa features an unusually long tail of 440 nm, which corresponds with the large size of the tape measure protein (3123 aa) [37], [38] and the protein is disproportionately large in comparison to other sequenced bacteriophages. The large unknown gene 221 likely encodes for a tail fiber component, with the C-terminus featuring significant homologies to Cellobiosidase, S-layer associated endoglucanase or glycoside hydrolase domains. The Tsamsa endolysin (gp217) is accompanied by a holin (gp215) and is active when produced recombinantly. The enzyme shows a broad lytic spectrum and lysed more *B. cereus* and *B. thuringiensis* strains than the phage infects. Thus the Tsamsa lysin might be useful as an antimicrobial agent against some *Bacillus cereus* sensu lato organisms.

In conclusion, we present vB_BanS-Tsamsa, a novel temperate phage obtained from *B. anthracis* that is specific to some members of the *Bacillus cereus* sensu lato group. To our knowledge Tsamsa is the largest sequenced siphovirus infecting *Bacillus* organisms.

## Supporting Information

Table S1 Database matches of predicted proteins encoded by *Bacillus anthracis* phage Tsamsa.
(DOCX)

## References

[1] Carter KC (1988) The Koch-Pasteur dispute on establishing the cause of anthrax. Bull Hist Med 62: 42–57. 3285924

[2] Okstad ØA, Kolstø A-B (2011) Genomics of *Bacillus* Species. In: Wiedmann M, Zhang W, editors. Genomics of Foodborne Pathogens. New Yorl: Springer. pp. 29–55.

[3] Drobniewski FA (1993) *Bacillus cereus* and related species. Clin Microbiol Rev 6: 324–338. 8269390 10.1128/cmr.6.4.324 PMC358292

[4] Soberon M, Pardo-Lopez L, Lopez I, Gomez I, Tabashnik BE, et al (2007) Engineering modified Bt toxins to counter insect resistance. Science 318: 1640–1642. 17975031 10.1126/science.1146453

[5] Stromsten NJ, Benson SD, Burnett RM, Bamford DH, Bamford JK (2003) The *Bacillus thuringiensis* linear double-stranded DNA phage Bam35, which is highly similar to the *Bacillus cereus* linear plasmid pBClin15, has a prophage state. J Bacteriol 185: 6985–6989. 14617663 10.1128/JB.185.23.6985-6989.2003 PMC262720

[6] Rasko DA, Altherr MR, Han CS, Ravel J (2005) Genomics of the *Bacillus cereus* group of organisms. FEMS Microbiol Rev 29: 303–329. 15808746 10.1016/j.femsre.2004.12.005

[7] Read TD, Peterson SN, Tourasse N, Baillie LW, Paulsen IT, et al (2003) The genome sequence of *Bacillus anthracis* Ames and comparison to closely related bacteria. Nature 423: 81–86. 12721629 10.1038/nature01586

[8] Buck CA, Anacker RL, Newman FS, Eisenstark A (1963) Phage Isolated from Lysogenic *Bacillus anthracis* . J Bacteriol 85: 1423–1430. 14047240 10.1128/jb.85.6.1423-1430.1963 PMC278351

[9] Schuch R, Fischetti VA (2009) The secret life of the anthrax agent *Bacillus anthracis*: bacteriophage-mediated ecological adaptations. PLoS One 4: e6532. 19672290 10.1371/journal.pone.0006532 PMC2716549

[10] Saile E, Koehler TM (2006) *Bacillus anthracis* multiplication, persistence, and genetic exchange in the rhizosphere of grass plants. Appl Environ Microbiol 72: 3168–3174. 16672454 10.1128/AEM.72.5.3168-3174.2006 PMC1472387

[11] Turner WC, Imologhome P, Havarua Z, Kaaya GP, Mfune JK, et al (2013) Soil ingestion, nutrition and the seasonality of anthrax in herbivores of Etosha National Park. Ecosphere 4: art13.

[12] Beyer W, Bellan S, Eberle G, Ganz HH, Getz WM, et al (2012) Distribution and molecular evolution of bacillus anthracis genotypes in Namibia. PLoS Negl Trop Dis 6: e1534. 22413024 10.1371/journal.pntd.0001534 PMC3295808

[13] Sambrook J, Russell DW (2001) Molecular Cloning - A Laboratory Manual. New York: Cold Spring Harbor Laboratory Press.

[14] Van Twest R, Kropinski AM (2009) Bacteriophage enrichment from water and soil. Bacteriophages: Springer. 15–21. 10.1007/978-1-60327-164-6_2 19066806

[15] Klumpp J, Dorscht J, Lurz R, Bielmann R, Wieland M, et al (2008) The Terminally Redundant, Nonpermuted Genome of *Listeria* Bacteriophage A511: a Model for the SPO1-Like Myoviruses of Gram-Positive Bacteria. J Bacteriol 190: 5753–5765. 18567664 10.1128/JB.00461-08 PMC2519532

[16] Adams MH (1959) Methods of study of bacterial viruses. Bacteriophages. New York: Interscience publishers, Inc. 443–457.

[17] Battisti L, Green BD, Thorne CB (1985) Mating System for Transfer of Plasmids among *Bacillus anthracis*, *Bacillus cereus*, and *Bacillus thuringiensis* . Journal of Bacteriology 162: 543–550. 3988702 10.1128/jb.162.2.543-550.1985 PMC218882

[18] Thorne CB (1968) Transducing bacteriophage for *Bacillus cereus* . Journal of virology 2: 657–662. 4972780 10.1128/jvi.2.7.657-662.1968 PMC375670

[19] Yamamoto KR, Alberts BM, Benzinger R, Lawhorne L, Treiber G (1970) Rapid bacteriophage sedimentation in the presence of polyethylene glycol and its application to large-scale virus purification. Virology 40: 734–744. 4908735 10.1016/0042-6822(70)90218-7

[20] Aziz RK, Bartels D, Best AA, DeJongh M, Disz T, et al (2008) The RAST Server: rapid annotations using subsystems technology. BMC Genomics 9: 75. 18261238 10.1186/1471-2164-9-75 PMC2265698

[21] Edgar RC (2004) MUSCLE: a multiple sequence alignment method with reduced time and space complexity. BMC Bioinformatics 5: 113. 15318951 10.1186/1471-2105-5-113 PMC517706

[22] Huelsenbeck JP, Ronquist F (2001) MRBAYES: Bayesian inference of phylogenetic trees. Bioinformatics 17: 754–755. 11524383 10.1093/bioinformatics/17.8.754

[23] Ronquist F, Huelsenbeck JP (2003) MrBayes 3: Bayesian phylogenetic inference under mixed models. Bioinformatics 19: 1572–1574. 12912839 10.1093/bioinformatics/btg180

[24] Pastagia M, Schuch R, Fischetti VA, Huang DB (2013) Lysins: the arrival of pathogen-directed anti-infectives. Journal of medical microbiology 62: 1506–1516. 23813275 10.1099/jmm.0.061028-0

[25] Keim P, Price L, Klevytska A, Smith K, Schupp J, et al (2000) Multiple-locus variable-number tandem repeat analysis reveals genetic relationships within *Bacillus anthracis* . Journal of Bacteriology 182: 2928–2936. 10781564 10.1128/jb.182.10.2928-2936.2000 PMC102004

[26] Fulmer P (2006) *Bacillus anthracis*: Antibiotic and Phage Sensitivity and Resistances: Louisiana State University.

[27] Klumpp J, Lavigne R, Loessner MJ, Ackermann HW (2010) The SPO1-related bacteriophages. Arch Virol 155: 1547–1561. 20714761 10.1007/s00705-010-0783-0

[28] Marti R, Zurfluh K, Hagens S, Pianezzi J, Klumpp J, et al.. (2013) Long tail fibers of the novel broad host range T-even bacteriophage S16 specifically recognize Salmonella OmpC. Mol Microbiol. 10.1111/mmi.12134 23289425

[29] Fouts DE, Rasko DA, Cer RZ, Jiang L, Fedorova NB, et al (2006) Sequencing *Bacillus anthracis* typing phages gamma and cherry reveals a common ancestry. J Bacteriol 188: 3402–3408. 16621835 10.1128/JB.188.9.3402-3408.2006 PMC1447464

[30] Minakhin L, Semenova E, Liu J, Vasilov A, Severinova E, et al (2005) Genome sequence and gene expression of *Bacillus anthracis* bacteriophage Fah. J Mol Biol 354: 1–15. 16226766 10.1016/j.jmb.2005.09.052

[31] Hendrix RW (2009) Jumbo bacteriophages. Curr Top Microbiol Immunol 328: 229–240. 19216440 10.1007/978-3-540-68618-7_7

[32] Smith MC, Thorpe HM (2002) Diversity in the serine recombinases. Mol Microbiol 44: 299–307. 11972771 10.1046/j.1365-2958.2002.02891.x

[33] Kilcher S, Loessner MJ, Klumpp J (2010) *Brochothrix thermosphacta* bacteriophages feature heterogeneous and highly mosaic genomes and utilize unique prophage insertion sites. J Bacteriol 192: 5441–5453. 20709901 10.1128/JB.00709-10 PMC2950505

[34] Kelly G, Prasannan S, Daniell S, Fleming K, Frankel G, et al (1999) Structure of the cell-adhesion fragment of intimin from enteropathogenic *Escherichia coli* . Nat Struct Biol 6: 313–318. 10201396 10.1038/7545

[35] Fraser JS, Yu Z, Maxwell KL, Davidson AR (2006) Ig-like domains on bacteriophages: a tale of promiscuity and deceit. J Mol Biol 359: 496–507. 16631788 10.1016/j.jmb.2006.03.043

[36] Barr JJ, Auro R, Furlan M, Whiteson KL, Erb ML, et al (2013) Bacteriophage adhering to mucus provide a non-host-derived immunity. Proc Natl Acad Sci U S A 110: 10771–10776. 23690590 10.1073/pnas.1305923110 PMC3696810

[37] Katsura I (1987) Determination of bacteriophage lambda tail length by a protein ruler. Nature 327: 73–75. 2952887 10.1038/327073a0

[38] Katsura I (1990) Mechanism of length determination in bacteriophage lambda tails. Adv Biophys 26: 1–18. 2150582 10.1016/0065-227x(90)90004-d

[39] Green BD, Battisti L, Koehler TM, Thorne CB, Ivins BE (1985) Demonstration of a capsule plasmid in *Bacillus anthracis* . Infect Immun 49: 291–297. 3926644 10.1128/iai.49.2.291-297.1985 PMC262013

