# Supplementary material for: Novel Giant Siphovirus from Bacillus anthracis Features Unusual Genome Characteristics
Source: PLoS One. 2014 Jan 27;9(1):e85972. doi: 10.1371/journal.pone.0085972 (PMC3903500; doi:10.1371/journal.pone.0085972)
Supplement: Table S1 — Database matches of predicted proteins encoded by Bacillus anthracis phage Tsamsa. (DOCX) [file pone.0085972.s001.docx]

**Table S1**: Database matches of predicted proteins encoded by *Bacillus anthracis* phage Tsamsa. Gene product is abbreviated as gp.

| **ORF** | **Best homology (BlastP e-value)** |
| --- | --- |
| gp13 | Serine/Threonine protein kinase (7.26E-7) |
| gp22 | M28/M42 family peptidase (6.58E-55) |
| gp63 | Methyltransferase (1.72E-70) |
| gp71 | Nicotinamide riboside transporter pnuC (4.26E-78) |
| gp73 | Nicotinamide riboside adenylyltransferase (2.69E-127) |
| gp77 | gp248 (*Bacillus* phage G) (5.35E-66) |
| gp79 | hypothetical protein, gp1.2 (Bacillus phage SPO1) (5.26E-72) |
| gp89 | Zink-dependent metalloprotease (2.37E-22) |
| gp92 | DNA-binding protein (6.57E-6) |
| gp94 | putative integrase/ site-specific recombinase (6.33E-45) |
| gp97 | Phage tail-like protein (1.15E-4) |
| gp98 | ATP/GTP binding protein (1.60E-4) |
| gp99 | L-alanoyl-D-glutamate peptidase (2.27E-33) |
| gp100 | SPBc2 prophage derived protein YorR (4.45E-33) |
| gp102 | Putative replicative DNA helicase (1.46E-141) |
| gp103 | Putative DNA primase (1.95E-91) |
| gp107 | Single stranded-DNA-specific exonuclease (8.42E-114) |
| gp109 | GTP pyrophosphokinase (3.95E-51) |
| gp114 | Appr-1-p processing protein (1.22E-42) |
| gp119 | Excinuclease ABC subunit C (4.79E-21) |
| gp136 | Putative HNH Endonuclease (5.20E-20) |
| gp137 | Transcription regulator (5.68E-26) |
| gp144 | DNA topoisomerase (6.66E-149) |
| gp145 | type IIA topoisomerase (0.00) |
| gp146 | PcfJ-like protein (3.14E-21) |
| gp148 | Ribonucleotide reductase (3.96E-36) |
| gp150 | Ribonucleoside-diphosphate reductase subunit (0.00) |
| gp151 | Ribonucleoside-diphosphate reductase subunit (1.11E-134) |
| gp152 | Thioredoxin/Glutaredoxin (8.85E-5) |
| gp153 | dUTP pyrophophatase (2.23E-36) |
| gp158 | Guanylate kinase (3.41E-29) |
| gp164 | Metal-dependent hydrolase (8.17E-81) |
| gp170 | gp109 (*Bacillus* phage G1) (1.60E-54) |
| gp173 | Extracellular protease/peptidase (4.49E-67) |
| gp174 | RNA ligase (1.21E-85) |
| gp175 | Phosphohydrolase (9.82E-56) |
| gp177 | ATP-dependent Clp endopeptidase subunit (2.92E-37) |
| gp178 | ATP-dependent DNA ligase (5.78E-97) |
| gp179 | RNA ligase (4.69E-73) |
| gp187 | gp66 (*Bacillus* phage G) (1.40E-28) |
| gp188 | Ribose-phosphate pyrophosphokinase (2.04E-70) |
| gp189 | Nicotinamide phosphoriboslytransferase (2.04E-153) |
| gp191 | gatB/qey domain protein (7.29E-16) |
| gp194 | gp153 (*Listeria* phage P100) (4.15E-13) |
| gp198 | Thymidylate synthase (6.42E-112) |
| gp200 | Dihydrofolate reductase (9.94E-32) |
| gp201 | ATP-dependent Clp protease subunit (3.06E-19) |
| gp202 | Depxoribonucleoside 5'-monophosphate phosphatase (4.06E-23) |
| gp203 | Thymidine kinase (2.54E-30) |
| gp205 | DNA polymerase III subunit alpha (0.00) |
| gp206 | Crossover junction endodeoxyribonuclease (1.27E-11) |
| gp213 | S-layer domain protein (2.20E-5), Ig-domain containing |
| gp215 | Phage holin (2.06E-6) |
| gp217 | Endolysin (1.21E-103) |
| gp220 | Minor structural protein (0.00) |
| gp221 | Putative tail fiber protein (4.44E-96) |
| gp222 | Putative tail fiber component, YkqB-like (6.79E-86) |
| gp223 | Minor structural protein (0.00) |
| gp226 | Tail tape measure protein (2.56E-155) |
| gp227 | Phage integrase/recombinase (3.74E-114) |
| gp233 | Phage structural protein, Ig-domain containing (9.96E-08) |
| gp235 | Structural protein (8.73E-17) |
| gp237 | Structural protein (3.25E-68) |
| gp239 | Structural protein (2.39E-36) |
| gp240 | Structural protein (4.79E-124) |
| gp241 | Terminase, ATPase subunit (0.00) |
| gp244 | DNA polymerase (1.81E-55) |
| gp248 | PhoH-family protein, ATP-binding (2.57E-19) |
| gp249 | DNA-binding protein (2.22E-05) |
| gp255 | Phage integrase (3.26E-16) |
| gp256 | gp27.3 (*Bacillus* phage SPO1) (9.19E-13) |
| gp257 | gp27.4 (*Bacillus* phage SPO1) (4.17E-40), ATP binding protein |
| gp259 | Peptidase M23 (3.64E-25) |
| gp264 | RecD/TraA family helicase (0.00) |
| gp267 | gp1.3 (*Bacillus* phage SPO1) (2.00E-06) |
